# The Impact of Different Types of Assistive Devices on Gait Measures and Safety in Huntington's Disease

**DOI:** 10.1371/journal.pone.0030903

**Published:** 2012-02-17

**Authors:** Anne D. Kloos, Deborah A. Kegelmeyer, Susan E. White, Sandra K. Kostyk

**Affiliations:** 1 Division of Physical Therapy, The Ohio State College of Medicine, The Ohio State University, Columbus, Ohio, United States of America; 2 Division of Health Information Management and Systems, The Ohio State College of Medicine, The Ohio State University, Columbus Ohio, United States of America; 3 Department of Neurology, The Ohio State College of Medicine, The Ohio State University, Columbus Ohio, United States of America; 4 Department of Neuroscience, The Ohio State College of Medicine, The Ohio State University, Columbus, Ohio, United States of America; Cardiff University, United Kingdom

## Abstract

**Background:**

Gait and balance impairments lead to frequent falls and injuries in individuals with Huntington's disease (HD). Assistive devices (ADs) such as canes and walkers are often prescribed to prevent falls, but their efficacy is unknown. We systematically examined the effects of different types of ADs on quantitative gait measures during walking in a straight path and around obstacles.

**Methods:**

Spatial and temporal gait parameters were measured in 21 subjects with HD as they walked across a GAITRite walkway under 7 conditions (i.e., using no AD and 6 commonly prescribed ADs: a cane, a weighted cane, a standard walker, and a 2, 3 or 4 wheeled walker). Subjects also were timed and observed for number of stumbles and falls while walking around two obstacles in a figure-of-eight pattern.

**Results:**

Gait measure variability (i.e., coefficient of variation), an indicator of fall risk, was consistently better when using the 4WW compared to other ADs. Subjects also walked the fastest and had the fewest number of stumbles and falls when using the 4WW in the figure-of-eight course. Subjects walked significantly slower using ADs compared to no AD both across the GAITRite and in the figure-of-eight. Measures reflecting gait stability and safety improved with the 4WW but were made worse by some other ADs.

## Introduction

Considerable resources are spent on the provision of assistive devices (ADs) for individuals with gait disturbances related to neurologic disorders. However, there are no evidence-based guidelines available upon which to base prescribing recommendations. Devices that do not meet the needs of individuals are unlikely to be used. In one survey, individuals with multiple sclerosis were noted to have abandoned ADs 30% of the time because of non-acceptance and 24.2% of the time because of inappropriate device recommendation [1]. Potential risks associated with the prescription of ADs in Parkinson's disease such as worsening of freezing during gait have been noted [2]. Different neurologic populations exhibiting distinct gait patterns are likely to have different needs and responses to ADs.

Gait impairments [3]–[7] and decreased postural stability [8], [9] lead to balance loss and falls in individuals with Huntington's Disease (HD) [7]. Gait impairments demonstrated in subjects with HD compared to age-matched controls include slower gait speed, shorter and more variable stride length, a wide base of support, decreased cadence, greater variability in stride time, swing time, and double support time, and increased trunk sway both in the anterior-posterior and medio-lateral directions [3]–[6], [10]. Subjects with HD also have greater postural sway in standing and demonstrate delayed motor responses to unexpected balance disturbances [8], [9]. The greater variation in spatial and temporal gait measures and increased postural sway are thought to be related to impaired/disordered processing of sensory feedback [11]. These balance and gait disorders lead to functional decline and increase the risk for falls in individuals with HD.

Loss of independent walking is the greatest predictor of nursing home placement in HD making treatment of gait disorders and fall prevention essential aspects of care for affected individuals [12]. Clinicians typically prescribe AD's such as canes and walkers in the belief that AD's will augment balance and prevent falls. To date there is little research to support this belief or to guide clinicians in their choices. AD selection should depend on objective assessments of a person's functional requirements and physical capabilities [13]. Therefore, we compared spatial and temporal gait measures while walking in a straight path and while maneuvering around obstacles with and without using different ADs. Based on previous findings in other patient populations and our own observations regarding the effects of ADs on gait characteristics [13]–[17], we hypothesized that the spatial and temporal gait measures would be: 1) different when subjects ambulated with an AD compared to without; 2) improved when subjects ambulated with a walker with swivel wheels (i.e., three-wheeled or four-wheeled) compared to walkers without swivel wheels (i.e., standard and two-wheeled), and 3) improved when subjects ambulated with a heavier cane compared to a standard cane. We also hypothesized that gait speed would be improved and there would be fewer losses of balance (i.e., stumbles or falls) during figure-of-eight turns when subjects used walkers with swivel wheels compared to no AD and other devices (i.e., canes and walkers without swivel wheels). The identification of ADs that are effective will enable clinicians to make more appropriate AD prescriptions for individuals with HD.

## Methods

### Ethics Statement

The study was approved by the Ohio State University Institutional Review Board. Written informed consent was obtained from all subjects participating in the study.

### Participants

Twenty-one volunteers were recruited from the Huntington's Disease Center of Excellence at the Ohio State University Medical Center. Inclusion criteria were a clinical diagnosis of Huntington's disease confirmed by a neurologist; ability to comprehend complex instructions as documented by ability to appropriately follow instructions needed to perform the standard UHDRS neuropsychiatric cognitive tests; ability to walk a minimum of 10 meters without an AD or physical assistance; absence of any additional central nervous system disorders; and absence of orthopedic and peripheral neurological disorders affecting the lower extremities. Individuals with HD have abnormal gait patterns compared to healthy individuals. The purpose of this study was to examine the effects of AD use on gait in individuals with HD; therefore subjects were used as their own controls with the no AD condition as the comparison or baseline condition.

### Instrumentation

**Figure 1:**
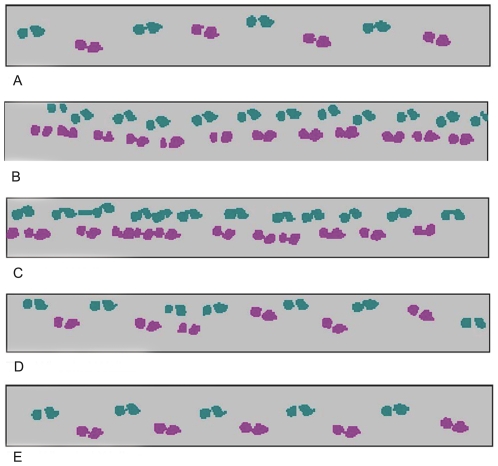
Footfall Patterns. Footfall pattern recordings in one individual with HD under five conditions (A) no assistive device and using a (B) cane, (C) standard walker, (D) two-wheeled walker or (E) four-wheeled walker. The four-wheeled walker (E) produced a gait pattern with the least variability.

Spatial and temporal measures of gait were collected using the GAITRite System® (CIR systems, Inc.: Havertown PA), a 4.88 m electronic walkway with sensors arranged in a gridlike pattern to capture footfall contacts. The application software (version 3.9) processes the raw data into footfall patterns (see Figure 1) and computes spatial and temporal parameters. The GAITRite measures are valid and reliable in subjects with HD [18], [19]. A standard aluminum straight cane with offset handle (Harvey Surgical Supply Corp., Flushing, NY), a heavy straight cane with offset handle weighing 1 pound (Harvey Surgical Supply Corp., Flushing, NY), a standard adult walker (StW; Graham-Field Health Products, Inc., Atlanta, GA), a two-wheeled walker (2WW) with fixed front wheels (Medline Industries, Inc., Mundelein, IL), a three-wheeled walker (3WW; Medline Industries, Inc., Mundelein, IL) and a four -wheeled walker with front swivel casters (4WW; Invacare Corporation, Elyria, OH) were utilized. All ADs were adjusted by researchers who are licensed physical therapists to fit subject height. These devices were chosen as they are the devices most frequently used by individuals with HD who attend our clinic. Both the 3WW and the 4WW were included as it would not be valid to assume that these two devices function equivalently given their very different designs (i.e., triangular versus square) and both are popular devices in our clinic population.

### Procedure

The UHDRS motor section was administered by a trained investigator (SK) and demographic data including age, sex, and number of years since symptom onset was obtained. Subjects reported whether they had experienced any falls in the past 6 months, with a fall defined as unintentionally coming to rest on the ground or other surface [20]. Leg length was measured as the distance from the greater trochanter to the bottom of the heel of the subject's footwear so that spatial measures were normalized to each subject's height.

Prior to testing, a therapist trained each subject on the use of the AD to be tested until the subject was observed to correctly and safely use the device. Training time for each device was equivalent to the time typically spent in our clinic to educate patients on device use. Subjects then walked at a normal, comfortable pace across the GAITRite walkway. Each subject performed 4 trials using no AD and with each of the 6 different ADs. The first trial under each condition was a practice trial. The GAITRite software averaged the data from the remaining three trials for each condition. Subjects were instructed to begin walking 2 meters before and stop 2 meters beyond the edges of the walkway to allow for acceleration and deceleration phases.

To test maneuverability of the different ADs around obstacles, subjects were timed using a stopwatch while they walked as fast as they could in a figure-of-eight pattern around two chairs set 4 feet apart under no AD and the 6 AD conditions. Each subject performed the figure-of-eight task twice and the time to complete the second trial was recorded. The investigators also recorded the number of observed stumbles (loss of balance from which the subject recovered without assistance) and falls (loss of balance for which the investigator provided assistance to prevent the subject from coming to the ground). The order of devices used was randomized and subjects were allowed to sit and rest before and between the GAITRite and figure-of-eight trials.

Coefficient of variation (CV) values were calculated for step time, stride length, swing time and double support time to assess the variability of gait measures across devices. For CV the average time series of steps across 3 walkway trials was utilized to calculate the mean and SD. Data for each of the gait measures and CVs were analyzed using one-way repeated-measures ANOVA to detect differences between the different walking conditions. Multiple comparisons were adjusted for through use of post-hoc Tukey tests. Significance was set a priori at <0.05. All statistical analysis was performed using SAS Version 9.2.

## Results

Subjects were on average 49.3±11 (25–66, range) years old, were 4.7±3.9 (1–14) years post clinical diagnosis, had Total Functional Capacity scores averaging 8±2.12 (4–11), had a mean CAG repeat size of 44.05±5.16 (37–58) and had Unified Huntington's Disease Rating Scale (UHDRS) motor sub-section scores of 40.4±14.4 (11–62) [21]. No subjects regularly utilized an AD at the time of the study. All subjects exhibited gait and balance deficits on the UHDRS and the GAITRite. Eight of the twenty-one subjects (38%) reported having fallen at least once in the last 6 months.

Canes and walkers are sometimes weighted to improve handling [21]. However, since heavy cane use did not alter gait measures as compared to the standard cane, heavy cane data was excluded from analysis. Therefore, only results for no AD and the 5 remaining devices are reported.

### Gait measures across different assistive devices

**Figure 2:**
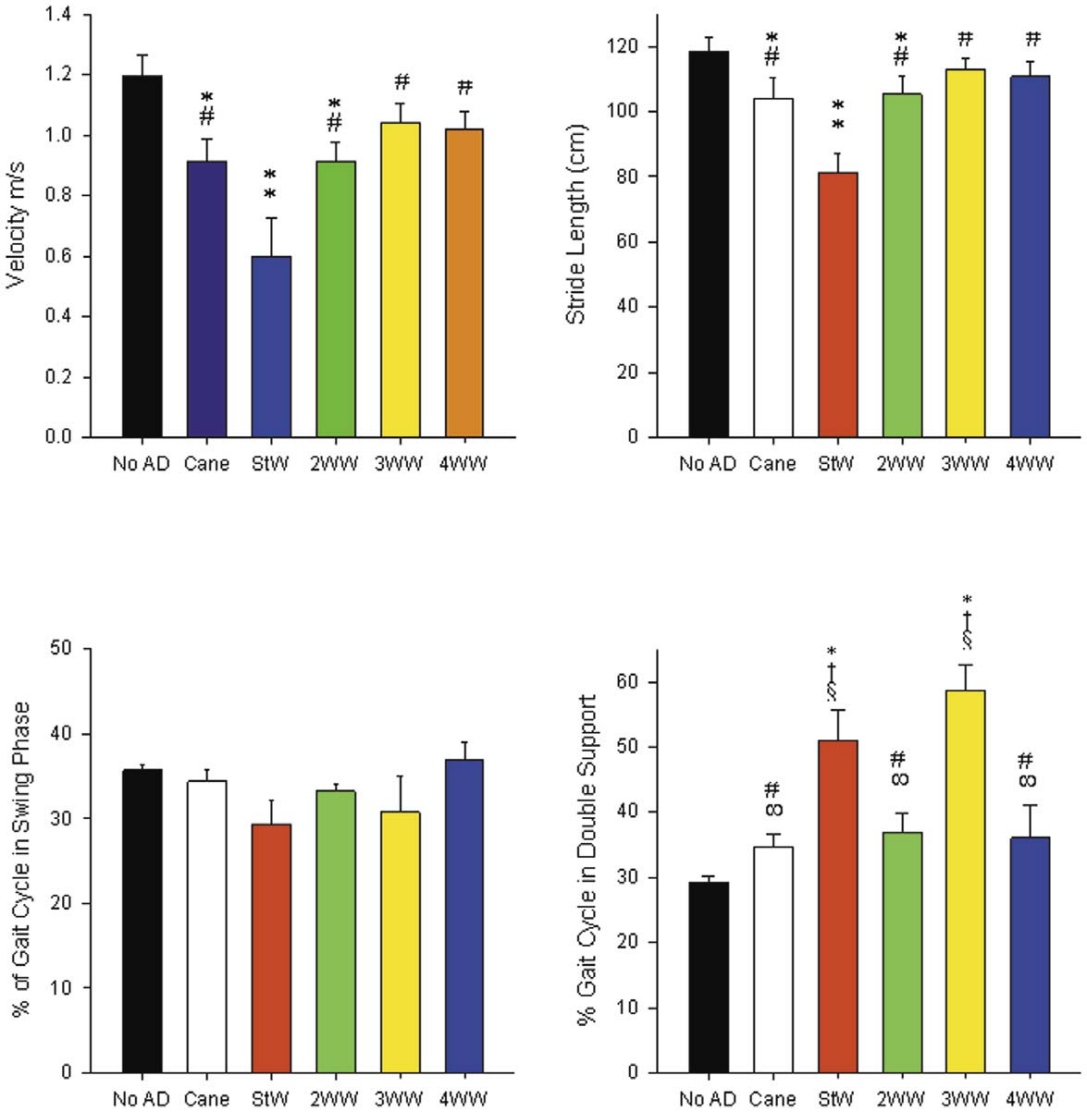
Gait Parameters. Comparison of gait parameters: (A) velocity, (B) stride length, (C) percent time in swing, and (D) percent time in double support with standard deviation across 6 walking conditions: no AD, no assistive device; StW, standard walker; 2WW, two-wheeled walker; 3WW, three-wheeled walker; 4WW, four-wheeled walker. * * significantly different than no AD at p<.05; **significantly different than all other conditions at p<.05; † significantly different than cane at p<.05; # significantly different than StW at p<.05; ∞ significantly different than 3WW at p<.05; § significantly different than 2WW, 4WW at p<.05.

Gait patterns varied markedly across the six conditions (Table 1, Figure 1). In comparison to the no AD condition, walking with ADs decreased mean velocity with the 4WW and 3WW being statistically equivalent to the no AD condition (Figures 1 and 2A–D). Walking with the 4WW and 3WW produced gait patterns with the highest velocity, longest stride length, and narrowest base of support (BOS) other than the no AD condition.In addition walking with the 4WW produced the lowest percent time in double support other than the no AD condition. In contrast, StW use produced the most dissimilar gait pattern with significantly decreased velocity and stride length and increased percent time spent in double support compared to no AD. The standard cane and 2WW also significantly reduced gait speed and stride length compared to no AD. Although subjects exhibited good velocity and stride length using the 3WW they had the highest percent time in double support (59%), which was significantly greater than no AD (29%; p<.05) and the 4WW (31%; p<.001). Walking with wheeled walkers produced a significantly narrowed base of support (BOS; p<0.05) compared with walking with no AD.

**Table 1:** Gait measures across all walking conditions: mean, (standard deviation).

	No AD	Cane	Standard Walker	2 Wheel Walker	3 Wheel Walker	4 Wheel Walker
Velocity (cm/s)	1.20 (0.07)	0.91* # (0.07)	0.60** (0.13)	0.91* # (0.06)	1.04# (0.06)	1.01# (0.06)
Stride Length (cm)	118.53 (4.37)	103.97* # (6.60)	81.46** (5.63)	105.50* # (5.29)	112.79# (3.5)	110.64# (4.71)
% Swing Time	35.7 (0.61)	34.38 (1.33)	29.32 (2.77)	33.23 (0.92)	30.75 (4.35)	36.84 (2.11)
% Double Support	29.18 (1.0)	34.66# ∞ (2.04)	51.01* † § (4.73)	36.9# ∞ (3.05)	58.88* † § (3.79)	31.07# ∞ (5.05)
Base of Support (cm)	12.75 (4.47)	13.5‡ (3.71)	11.52 (4.52)	10.58* † (3.21)	9.55* † (3.45)	10.59* † (3.44)

Abbreviations: no AD, no assistive device; StW, standard walker; 2WW, two wheeled walker; 3WW, three wheeled walker; 4WW, four wheeled walker; CV, Coefficient of Variation;

*significantly different than no AD at p<.05;

**significantly different than all other conditions at p<.05;

† significantly different than cane at p<.05;

# significantly different than StW at p<.05;

∞ significantly different than 3WW at p<.05;

‡ significantly different from 2WW, 3WW, 4WW at p<.05;

§ significantly different than 2WW, 4WW at p<.05.

### Gait measure variability across different walking conditions

**Figure 3:**
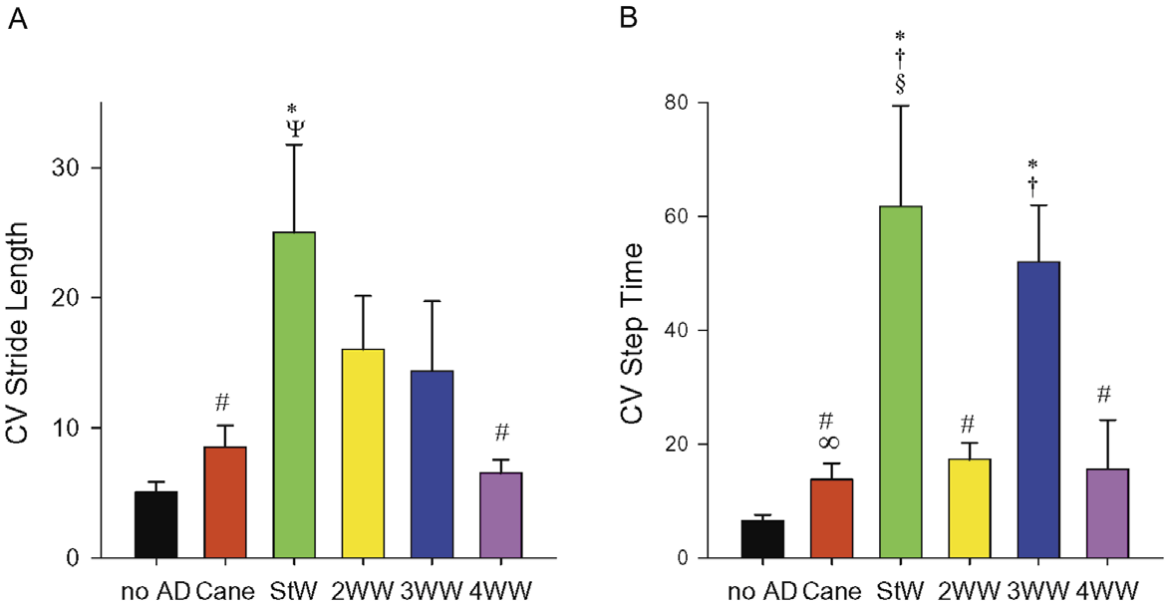
Coefficients of Variation. Comparison of mean coefficients of variation across six walking conditions: (A) step time and (B) stride length coefficients of variation (CV) with standard deviation. Variability was consistently low when using the four-wheeled walker (4WW); no AD, no assistive device; StW, standard walker; 2WW, two-wheeled walker; 3WW, three-wheeled walker; *significantly different than no AD at *p*<.05. * significantly different than no AD at p<.05; † significantly different than cane at p<.05; # significantly different than StW at p<.05; ∞ significantly different than 3WW at p<.05; § significantly different than 2WW, 4WW at p<.05; Ψ significantly different than cane and 4WW at p<.05.

Compared to the other devices, walking with the 4WW produced a gait pattern with the least variability in step to step measures. (Table 2/Figure 3A–B) Walking with the StW and the 3WW produced more variability in gait measures compared to no AD and several other devices (Table 1). Step time and stride length variability (i.e., CVs) were significantly (p≤.05) increased during walking with the StW (Table 2, Figure 3). Walking with the 3WW significantly increased (p≤.05) step time, swing time, and double support time variability (Table 2). Use of the2WW significantly increased (p≤.05) variability in step time and double support time. Across the devices, the 4WW consistently produced low gait measure variability.

**Table 2:** Coefficient of Variation of Gait measures across all walking conditions: mean, (standard deviation).

	No AD	Cane	Standard Walker	2 Wheel Walker	3 Wheel Walker	4 Wheel Walker
Base of Support (cm)	12.75 (4.47)	13.5‡ (3.71)	11.52 (4.52)	10.58* † (3.21)	9.55* † (3.45)	10.59* † (3.44)
Step Time CV	6.47 (1.12)	13.77# ∞ (2.82)	61.76* † § (17.67)	17.30# (2.88)	51.98* Ψ (9.96)	6.98# ∞ (8.64)
Stride Length CV	5.03 (0.82)	8.52# (1.66)	25.03* Ψ (6.78)	16.04 (4.10)	14.46 (5.39)	6.62# (1.03)
Swing Time CV	8.94 (1.51)	27.91 (15.29)	54.24 (20.95)	19.20∞ (2.94)	73.49* § (12.66)	8.09∞ (9.41)
Double Support Time CV	9.51 (0.64)	19.99∞ (3.86)	31.89* 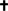 (4.45)	29.27* 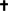 (5.96)	38.06* Ψ (4.83)	11.42 # 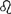 (10.52)

Abbreviations: no AD, no assistive device; StW, standard walker; 2WW, two wheeled walker; 3WW, three wheeled walker; 4WW, four wheeled walker; CV, Coefficient of Variation;

*significantly different than no AD at p<.05;

† significantly different than cane at p<.05;

# significantly different than StW at p<.05;

∞ significantly different than 3WW at p<.05;

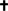
significantly different from 4WW at p<.05;

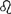
significantly different from 2WW and 3WW at p<.05;

‡ significantly different from 2WW, 3WW, 4WW at p<.05;

§ significantly different than 2WW, 4WW at p<.05;

Ψ significantly different than cane and 4WW at p<.05.

### Gait when maneuvering through a figure-of-eight

**Figure 4:**
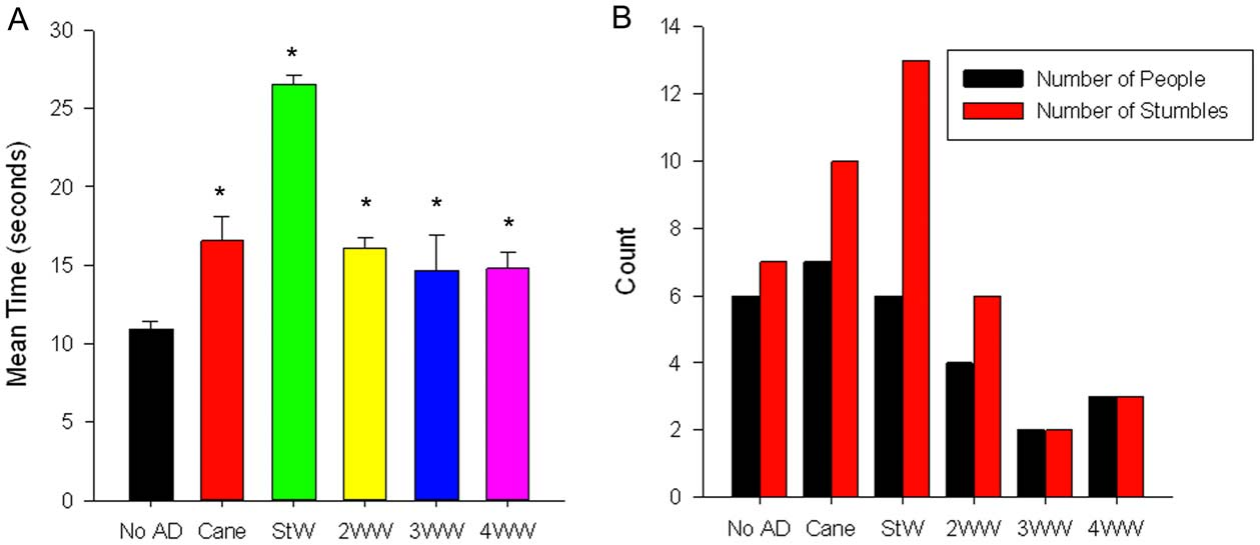
Figure of Eight Course. Comparison of mean time and stumbles on a figure of eight course: (A) mean time with standard deviation for one lap around figure-of-eight course and (B) number of stumbles and number of individuals who stumbled walking in the figure-of-eight course across conditions; no AD, no assistive device; StW, standard walker; 2WW, two-wheeled walker; 3WW, three-wheeled walker; 4WW, four-wheeled walker. * significantly different than no AD at *p*<.001.

Walking speed was significantly faster with no AD than all devices (p<.001) while walking with a StW was significantly slower than all other conditions (p<.00001; Figure 4A). Use of the 4WW resulted in faster completion times than all other devices except the 3WW and was significantly faster than the 2WW (p<.05). The number of stumbles was highest with the StW. Stumbles were less common with the 3WW and 4WW (Figure 4B), and in fact were less frequent than with no AD. Three falls occurred in different subjects while using no AD, the StW and the 3WW. Bumping the chairs with the devices was not associated with stumbles or falls. Overall the 4WW performed better than any other device when maneuvering around obstacles.

## Discussion

Gait and mobility abnormalities significantly affect the independence and quality of life of individuals with HD [12]. Although ADs are routinely prescribed for neurological gait disorders, the effects of different ADs on gait patterns have not been previously analytically examined for specific neurological patient populations. We have systematically examined the effects of different ADs on spatial and temporal gait measures and maneuverability in individuals with HD. Our findings illustrate the significant impact that canes and walkers have on gait patterns of individuals with HD both during walking on a straight path and around obstacles. The 4WW produced a gait pattern with the least variability and with the least impact on the individual's ability to ambulate at their usual walking speed. In addition, use of the 4 WW resulted in a gait pattern that was safer than with no AD with reduced stumbles and falls.

Compared to other devices, the 4WW produced a gait pattern that was more similar to each individual's spontaneous gait pattern without an AD but with less variability and more stability. This finding concurs with a study by Alkjaer et al. [17] which showed that healthy middle aged females walked at equal speeds with no AD and with a 4WW. Improved gait measures with the 4WW over no AD included a narrowing of the base of support and increased percent time in the swing phase. It is also noteworthy that those using the 4WW had fewer stumbles and falls during figure-of-eight walking than when not using an AD. Greater stability of the 4WW due to a wider base of support and more support during turning than canes, Stw and no AD may underlie these improvements. The 4WW also appears to provide greater ease of use as it allows the individual to simply apply pressure with the hands to propel it. The cane, StW, and 2WW require the user to lift the device in time with their stepping whereas the 3WW and 4WWs allow the person to push the device without lifting it. Ease of use is a concern when prescribing ADs for individuals with HD who have difficulties with learning sequences of movements and performing a second task during walking [22]. Our observation that subjects generally took longer to learn how to use the cane and StW compared to the wheeled walkers would support this statement.

Gait with the 3WW was equivalent to the 4WW across several measures but subjects spent more time in the stance phase with this device than any other device and had significantly increased gait variability. Increased stance time is a compensatory strategy that people with gait instability often adopt to prevent falls [23], [24]. Thus, the triangular design of the 3WW may provide less medial-lateral stability than other wheeled walkers leading to unsteadiness and increased stance time. This may be an important consideration when prescribing ADs for patients with HD who have increased trunkal sway related to chorea and dystonia. Other possible explanations for prolonged stance time and greater gait variability with 3WW use are that it's triangular shape makes maneuvering it a more challenging task cognitively or it's narrowed front causes individuals to alter their stepping patterns. Examination of kinematic data which was collected during this study but not yet analyzed may provide further insight.

The 2WW and StW produced the slowest gait speeds and shortest stride lengths compared to the other ADs when walking on a straight path. One explanation is that the smaller wheels on the 2WW roll less smoothly than those on the 3WW and 4WWs. Similar findings were reported by Mahoney et al. [14] who found that stride length was decreased and time to walk an obstacle course was increased with the 2WW as compared to the 3WW in elderly subjects. The 2WW must be picked up when turning or even when maintaining a straight path, whereas 3WW and 4WW's simply require the person to push on the devices. Individuals with HD change path directions and may have to maneuver the 2WW and StW more to keep going straight, thus explaining the slowing and increased variability (i.e., coefficient of variations) of gait even on the straight path. This is consistent with our finding that the 3WW and 4WW produced the highest velocities and stride lengths compared to other ADs.

The variability in gait measures was lower with the 4WW than any of the other devices. This may be clinically important, as higher variability has been shown to correlate with increased falls in the elderly [25], [26] and those with Parkinson disease [4]. The StW and 3WW's exhibited the highest variability across all measures followed by the 2WW. Canes performed better than the standard, 2W and 3W walkers but had higher variability in all measures than the 4WW. These findings indicate that subjects adopted a safer and less variable gait when utilizing the 4WW. Based on previous studies [25]–[27] low variability utilizing the 4WW would indicate a lower fall risk with this device than with the StW, canes, 2W and 3W walkers.

Because individuals with HD demonstrate improved grasp and arm movement when lifting and transporting a heavier object compared to a lighter one, presumably due to increased somatosensory feedback [28], we had hypothesized that using the heavier cane would affect gait patterns differently compared to the standard cane. However, we found no significant differences between use of a standard cane and a weighted cane on gait measures. One explanation for our finding is that the complexity of coordinating walking with movement of a cane was so challenging for many subjects that it overshadowed any benefits from the additional weight. More research is needed to determine whether weighting ADs improves gait patterns or function in individuals with HD.

Maneuverability is an important factor to consider when prescribing an AD as many individuals with HD fall when turning or avoiding obstacles in their paths. The figure-of-eight course utilized in this study appeared to be a sensitive measure of the ability to make turns and safely maneuver around objects during gait. As anticipated, subjects walked the fastest and had the fewest number of stumbles when using the 3WW and the 4WW compared to other ADs. This is not surprising since the walkers with front swivel and rear wheels allow turning without additional maneuvering of the device, whereas the 2WW must be picked up during turning maneuvers. Lack of support during turns may explain why there were more stumbles with the 2WW than either of the other wheeled walkers. Of the three recorded falls, one occurred with the 3WW, one with the StW, and one with no AD.These findings indicate that subjects were able to make turns and changes in direction in a more timely and safe manner with the 4WW.

This is the first study in any neurological patient population that systematically examines the effects of different ADs on spatial and temporal gait measures and maneuverability; however, there are several limitations to the study. Subjects in the study were not regular users of ADs and thus device use was a novel task for these individuals. However, subjects were trained on each device and allowed to practice until they exhibited mastery of proper technique and stated they felt comfortable using the device. Nonetheless, some aspects of gait performance must be assumed to be due to the novelty of utilizing a device. We attempted to control for this limitation in several ways: 1) order of device use was randomized across subjects and 2) novelty was a consistent factor across all devices and thus did not affect any one device more than another. Although subjects in this study were not regular AD users, it should be noted that the subjects' UHDRS motor scale and TFC scores, and the high number of fallers indicated that they had gait deviations that made them potential candidates to be assessed for assistive device use. In fact two subjects who were frequent fallers adopted the use of a 4WW immediately following the study. Another limitation was that devices were being utilized in an artificial environment rather than in a real world environment. This limits our ability to fully assess device performance across all possible aspects of use.

In conclusion, walking with a 4WW with front swivel wheels produced a more efficient, consistent and safe gait pattern than other commonly prescribed ADs in individuals with HD both on a straight path and during turns. The greater stability, ease of use, and maneuverability of the 4WW over other devices may account for its better performance. These features are likely to make the 4WW more acceptable to patients and increase likelihood that the device will be used. Unlike most gait disorders where increased age is associated with increased falls, younger individuals with HD motor symptoms tend to have a greater risk of falls than elderly patients with HD with the same degree of motor impairment [29]. These younger individuals are more reluctant to accept the use of an AD that might slow them down. Based on these findings, we recommend that clinicians consider prescribing 4WWs over other ADs for gait impairments and fall prevention for individuals with HD. Future studies to examine other aspects of device use such as performance in real world and outdoor environments are still needed.
